# Amelioration of Hippocampal Insulin Resistance Reduces Tau Hyperphosphorylation and Cognitive Decline Induced by Isoflurane in Mice

**DOI:** 10.3389/fnagi.2021.686506

**Published:** 2021-08-25

**Authors:** Liangyu Peng, Xin Fang, Fangxia Xu, Shuai Liu, Yue Qian, Xiangdan Gong, Xin Zhao, Zhengliang Ma, Tianjiao Xia, Xiaoping Gu

**Affiliations:** ^1^Department of Anesthesiology, Affiliated Drum Tower Hospital of Medical Department of Nanjing University, Nanjing, China; ^2^Medical School of Nanjing University, Nanjing, China; ^3^Department of Anesthesiology, Nanjing Stomatological Hospital, Medical School of Nanjing University, Nanjing, China; ^4^Jiangsu Key Laboratory of Molecular Medicine, Nanjing University, Nanjing, China

**Keywords:** cognitive impairment, isoflurane, insulin resistance, metformin, hippocampus, blood glucose

## Abstract

General anesthetics can induce cognitive impairments and increase the risk of Alzheimer’s disease (AD). However, the underlying mechanisms are still unknown. Our previous studies shown that long-term isoflurane exposure induced peripheral and central insulin resistance (IR) in adult mice and aggravated IR in type 2 diabetes mellitus (T2DM) mice. Clinical and preclinical studies revealed an association between impaired insulin signaling and tau pathology in AD and other tauopathies. We investigated if alleviation of hippocampal IR by the antidiabetic agent metformin could reduce tau hyperphosphorylation and cognitive decline induced by isoflurane in mice. The effects of prolonged (6 h) isoflurane anesthesia on hippocampal IR, hippocampal tau hyperphosphorylation, and hippocampus-dependent cognitive function were evaluated in wild type (WT) adult mice and the high-fat diet plus streptozotocin (HFD/STZ) mouse model of T2DM. Here we shown that isoflurane and HFD/STZ dramatically and synergistically induced hippocampal IR and fear memory impairment. Metformin pretreatment strongly ameliorated hippocampal IR and cognitive dysfunction caused by isoflurane in WT mice, but was less effective in T2DM mice. Isoflurane also induced hippocampal tau hyperphosphorylation and metformin reversed this effect. In addition, isoflurane significantly increased blood glucose levels in both adult and T2DM mice, and metformin reversed this effect as well. Administration of 25% glucose to metformin-pretreated mice induced hyperglycemia, but surprisingly did not reverse the benefits of metformin on hippocampal insulin signaling and fear memory following isoflurane anesthesia. Our findings show hippocampal IR and tau hyperphosphorylation contribute to acute isoflurane-induced cognitive dysfunction. Brief metformin treatment can mitigate these effects through a mechanism independent of glycemic control. Future studies are needed to investigate whether long-term metformin treatment can also prevent T2DM-induced hippocampal IR and cognitive decline.

## Introduction

About 10–50% of patients receiving surgery under anesthesia demonstrate cognitive impairments occurring between 1 month and 1 year after surgery following treatment, termed postoperative cognitive dysfunction (POCD) (James, 2020). POCD is not a transient phenomenon but associated with prolonged hospitalization, functional decline, less likely to live independently, and increased mortality (Moller et al., 1998; Steinmetz et al., 2009). Intraoperative hypoxia, inflammation, and the pharmacological effects of anesthetics are implicated in POCD pathogenesis, and older age is the strongest risk factor (Moller et al., 1998). Further, accumulating clinical and experimental evidence suggest that surgery under general anesthesia increases the risk of developing Alzheimer’s disease (AD) and other forms of dementia (James, 2020). Our previous studies confirmed that long-term isoflurane exposure can induce cognitive impairment in adult mice, providing an experimental model to investigate the underlying pathogenesis (Xia et al., 2016; Song et al., 2019).

In addition to increasing POCD risk, aging increases susceptibility to metabolic syndrome (MetS) and type 2 diabetes mellitus (T2DM) (Ford et al., 2002; Menke et al., 2015). It is also well accepted that MetS can induce cognitive impairments, including of memory and executive function (Kim and Feldman, 2015; Ng et al., 2016). Moreover, MetS increases the risk of POCD (Hudetz et al., 2011). A decline in brain tissue volume can be detected even in early onset MetS patients (Sala et al., 2014). Similarly, T2DM disrupts brain structure and function, especially in cognition-related regions, and increases the risks of both POCD and age-related cognitive decline compared to age-matched non-diabetics (Feinkohl et al., 2017; Sanjari Moghaddam et al., 2019). Our pilot study also found that T2DM predicts POCD at 1 week after orthopedic surgery in elderly patients (over 60 years of age) and patients with T2DM had a 2.6-fold higher risk of POCD compared with patients without diabetes at 1 week after surgery (Supplementary Table 1). Collectively, these observations suggest that the molecular signaling mechanisms disrupted in T2DM may also contribute to POCD.

A decrease in insulin-induced molecular signaling, termed insulin resistance (IR), is a major pathogenic mechanism in MetS and T2DM, and may contribute to the associated cognitive impairments (Biessels and Reagan, 2015; Kim and Feldman, 2015). Experimental models of diabetes not only show remarkable peripheral and central nervous system IR, but also deficits in the neuroplastic processes linked to cognition (Biessels and Reagan, 2015). Moreover, brain and peripheral IR increase with advanced age (Petersen et al., 2015; Martín-Segura et al., 2019). Insulin resistance is also a common complication after anesthesia and surgery, and the degree of postoperative IR is associated with surgical magnitude (Thorell et al., 1993). In aged rats as well, surgical procedures impair central insulin signaling and increase susceptibility to hippocampus-related cognitive impairments (Kawano et al., 2016). Isoflurane and sevoflurane reduced peripheral insulin sensitivity by about 50% and almost completely suppressed liver insulin responses in a canine model (Kim et al., 2016). These anesthetics also significantly reduced mean cerebral glucose utilization during anesthesia in rats (Lenz et al., 1998). Recently, we found that long-term isoflurane exposure induced peripheral and central IR in adult mice and aggravated IR in T2DM mice (Fang et al., 2020). Clinical studies show that preoperative IR predicts POCD in elderly gastrointestinal patients and that the IR index is significantly higher in patients with POCD than patients without POCD (Tang et al., 2017; He et al., 2019). Collectively, these results provide compelling evidence that IR contributes to POCD development.

Hippocampal accumulation of hyperphosphorylated tau protein may link MetS/T2DM, IR, POCD, and AD risk. Hyperphosphorylated tau proteins are a core component of neurofibrillary tangles, a pathological hallmark of AD (Ittner et al., 2010), and IR can prevent hippocampal tau dephosphorylation in rats (Kim et al., 2015). Both clinical and preclinical studies have found an association between impaired insulin signaling and tau pathology in AD and other tauopathies (Yarchoan et al., 2014). Further, inhalational anesthetic exposure, especially prolonged or repeated exposure, induced a long-lasting increase in tau phosphorylation associated with cognitive impairment (Dong et al., 2012; Le Freche et al., 2012). Conversely, insulin-sensitizing strategies have been shown to improve memory performance, reduce cerebrospinal fluid biomarkers of disease, and enhance cerebral glucose utilization in mild cognitive impairment (MCI) and AD patients (Hölscher, 2014).

Based on these findings, we speculated that isoflurane-induced hippocampal IR and ensuing tau hyperphosphorylation contribute to POCD and progression of AD pathology, while amelioration of hippocampal IR may mitigate tau hyperphosphorylation and POCD caused by long-term isoflurane anesthesia. Metformin, a first-line antidiabetic drug, exerts its therapeutic glucose-lowering effects by suppressing hepatic gluconeogenesis, decreasing insulin resistance, and increasing insulin sensitivity (Giannarelli et al., 2003). Here we examine whether pretreatment with metformin can alleviate hippocampal IR and tau hyperphosphorylation as well as cognitive impairment caused by long-term isoflurane anesthesia in adult and T2DM mice.

## Materials and Methods

### Animals and Treatment

**FIGURE 1:**
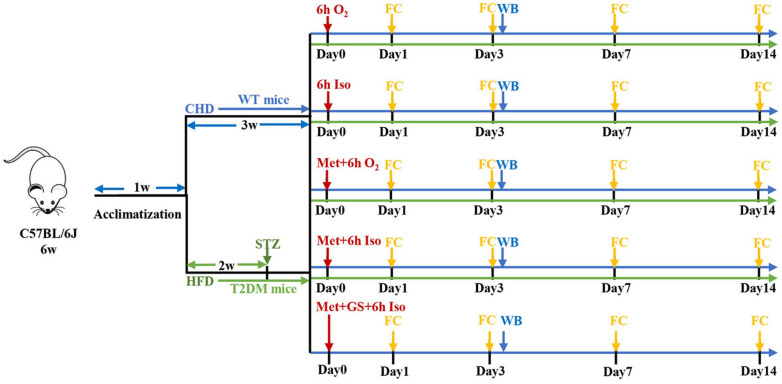
Schematic presentation of experimental design. CHD, chow diet; HFD, high-fat diet; STZ, streptozotocin; Iso, isoflurane; Met, metformin; GS, 25% glucose solution; FC, fear conditioning test; WB, Western blot.

Animal care and study protocols were approved by the Laboratory Animal Ethics Committee of Drum Tower Hospital. Animals were housed (6–7 per cage) with free access to food and water and were kept in temperature-controlled facilities at 22–25°C under a 12 h light/dark cycle. Diabetic mellitus type 2 (T2DM) mice model was prepared as we described previously (Fang et al., 2020). After 1 week of acclimatization, 7-week-old male C57BL/6J wild type (WT) mice from the Model Animal Research Center of Nanjing University were fed either chow diet or high-fat diet (HFD, 60% kcal fat, 20% carbohydrate). After fed with HFD for 2 weeks, inducible diabetic mellitus type 2 mice were fasted overnight and intraperitoneally injected with streptozotocin (STZ, Sigma-Aldrich, United States) freshly dissolved in 0.1 mM citrate buffer (pH 4.5) at a dose of 60 mg/kg body weight and tested for fasting 12 h tail vein blood glucose levels 1-week post-injection. Mice fed with chow diet were injected intraperitoneally with the citrate buffer vehicle. Mice with typical features of T2DM (polyphagia, polydipsia and polyuria) and fasting blood glucose levels more than 11.1 mmol/L were considered to be T2DM and were used in the experiment. Animals were maintained with their respective diets and body weight was measured every 3 days during the experiment. WT mice fed a standard chow diet were randomly divided into five subgroups receiving vehicle injection (Con), isoflurane anesthesia (Anes), metformin injection (Met), metformin pretreatment plus isoflurane anesthesia (Met + Anes), or metformin plus gavage 25% glucose solution prior to isoflurane (Glu + Met + Anes). Another group of T2DM model mice was randomly divided into a control group (DM), isoflurane anesthesia group (DM + Anes), metformin group (DM + Met), and metformin pretreatment plus isoflurane anesthesia group (DM + Met + Anes). Mice in Anes, Met + Anes, Glu + Met + Anes, DM + Anes, and DM + Met + Anes groups were exposed to isoflurane anesthesia in a chamber prefilled with 4% isoflurane (Lunan Better Pharmaceutical Co.) in 100% oxygen and then maintained with 1.3% isoflurane in 100% oxygen flowing at 2.5 L/min for 6 h. The respiratory activities of mice were monitored during anesthesia. To prevent the effect of hypothermia on tau protein phosphorylation, mice were placed on heating pads to maintain body temperature during anesthesia and lasted until full recovery. Mice in Met, Met + Anes, Glu + Met + Anes, DM + Met, and DM + Met + Anes groups were injected intraperitoneally with metformin (Sigma-Aldrich, D150959) 50 mg/kg body weight 1 h before anesthesia or at the corresponding time. Mice in the Glu + Met + Anes group were administered 25% glucose solution 0.1 ml (25%Glu 0.1 ml + Met + Anes) or 0.2 ml (25%Glu 0.2 ml + Met + Anes) by gavage before anesthesia (Figure 1).

### Measurements of Blood Glucose

To eliminate interference from cage mates, mice were housed singly during tail vein blood sampling. Experienced researchers cautiously snip 1–2 mm of tissue from the tail tip distal with sharp scissors, and blood was obtained by gently massaging the tail. Squeezing the tail violently should be avoided because it might be painful for the mice and affect the quality of the sample. Stroking over the incision gently with sterile swabs dipped in saline reopened it and several blood samples could be collected in subsequent experiments. Blood glucose levels were tested by a hand-held glucometer (ACCU-CHEK Active, Roche, Switzerland).

### Fear Conditioning Test

The fear conditioning test was conducted in 30 cm × 37 cm × 25 cm chambers equipped with a sound amplifier to produce auditory cues and a metal grid floor to deliver conditioning foot shocks. During the training phase, a mouse was placed in the chamber and allowed to explore freely for 3 min as habituation. After habitation, the sound (4,000 Hz, 100 dB) was presented for 30 s (conditioned stimulus, CS) and co-terminated with a foot shock (2 s, 0.8 mA) as an unconditional stimulus (US). Freezing behavior (a sign of conditioned fear) was scored for 1 min after foot shock. The chamber was then cleaned and descented with 75% ethanol between trials. Contextual fear conditioning was tested 24 h later in the same chamber by scoring freezing behavior (% of total time) for 3 min in the absence of sound or foot shock. A cued fear memory test was performed 2 h after the contextual fear conditioning test. Briefly, the sensory environment of the chamber (textures, odors, and colors) was modified and animals allowed 3 min of exploration as an accommodation period, followed by delivery of the CS (4,000 Hz, 100 db, 30 s). Freezing time (% of total time) was recorded automatically and the data were analyzed using Packwin 2.0 software.

### Real-Time Reverse Transcription Polymerase Chain Reaction (RT-PCR)

The total RNA of hippocampus was extracted using PureLink RNA mini kit (Biotek, RP1202) and cDNAs were synthesized using cDNA synthesis kit (TakaRa, RR036A). RNA quality and quantity were measured by ultraviolet spectroscopy (Biotek, United States). RT-PCR was performed using SYBR Premix Ex Taq (Takara, RR420A) in the ABI StepOne Plus Real-Time PCR system (Applied Biosystems). The primers (TSINGKE, China) were shown as follows: β-actin (F: 5′-CTGTCCCTGTATGCCTCTG-3′, R: 5′-ATGTCACGCACGATTTCC-3′). GSK3β (F: 5′-ATTCCCT CAAATTAAGGCACATCC-3′, R: 5′-ATACTCCAGCAGACG GCTACACAG-3′); AKT (F: 5′-TGCATTGCCGAGTCCAGA A-3′, R: 5′-GCATCCGAGAAACAAAACATCA-3′); IRS1 (F: 5′ -GTTTCCAGAAGCAGCCAGAG-3′, R: 5′-ACTCTCTCCAC CCAACGTGA-3′); IRS2 (F: 5′-CATCGACTTCCTGTCCC ATCA-3′, R: 5′-CCCATCCTCAAGGTCAAAGG-3′); Gene expression data were normalized to β-actin.

### Western Blotting

The expression of tau, NR2B and insulin-signaling proteins were detected in the hippocampus by western blotting. Tissues were washed with ice-cold PBS and then lysed in proteinase and phosphatase inhibitors (Sigma, United States) containing RIPA buffer. The samples were centrifuged at 12,000 rpm, 4°C for 20 min. BSA method was performed to determine the protein contents. Twenty to forty microgram proteins per lane were separated by electrophoresis in 8% SDS-PAGE gels (KayGen Biotech, Co., Ltd.) and blotted onto polyvinylidene difluoride membranes (PVDF; Bio-Rad Laboratories, United States). The membranes were blocked with 5% non-fat milk for 2 h at normal temperature and then were incubated with the following primary antibodies overnight at 4°C: anti-tau (phospho Ser396) (1:500, 9632S, CST), anti-tau (phospho Ser202 and Thr205, AT8) (1:500, MN1020, Invitrogen), anti-tau (1:500, BS3738, Bioworld technology), anti-NR2B (phospho Tyr1472) (1:500, ab3856, Abcam), anti-NR2B (1:500, ab65783, Abcam), anti-IRS1(phospho Ser636/639) (1:500,2388, CST), anti-IRS1 (phospho Tyr896) (1:500, ab46800, Abcam), anti-IRS1 (1:500,ab52167, Abcam), anti-IRS2 (phospho Ser731) (1:500, ab3690, Abcam), anti-IRS2 (1:500,4502, CST), anti-AKT (phospho Ser473)(1:1,000, 4060, CST), anti-AKT (1:1,000, 4685, CST), anti-β-actin (1:1,000, ab8226, Abcam), anti-GSK3β (1:1,000, 12456, CST), and anti-GSK3β (phospho Ser9)(1:1,000, 5558, CST). β-actin were used as loading controls, respectively. Antibodies were diluted with 5% Bovine serum albumin (BSA; Gentihold) solvents. The membranes were washed three times with TBST and then incubated with HRP-conjugated antibodies for 2 h. The proteins were visualized using a chemiluminescence kit (ECL; Pierce, Illinois, United States). Band densities of protein were quantified via ImageJ (National Institutes of Health, United States).

### Statistical Analysis

All data were parametric and summarized as mean ± standard deviation (SD). Results from cognitive behavioral tests, Western blotting, RT-PCR and light absorbance were analyzed by a one-way ANOVA test, followed by Bonferroni multiple comparison test. Changes in blood glucose levels over time were analyzed using multivariate analyses of variance for repeated measures followed by Bonferroni *post hoc* analysis. Statistical analysis was carried out using SPSS 25.0 software (IBM Corporation, Armonk, NY). Statistical significance referred to differences at the level of *P* < 0.05.

## Results

### Isoflurane and T2DM Synergistically Induce Hippocampus-Dependent Cognitive Dysfunction in Adult Mice

**FIGURE 2:**
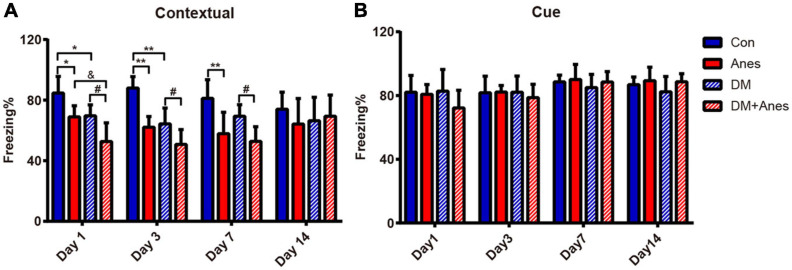
Isoflurane inhalation selectively impairs contextual fear memory and this effect is exacerbated in diabetic mice. **(A)** Freezing (% time) in response to the foot-shock context (chamber) among adult WT mice and T2DM mice (DM groups) exposed to 1.3% isoflurane inhalation for 6 h (Anes and DM + Anes groups) or control inhalation (100% O_2_ for 6 h). **(B)** Freezing time during exposure to the conditioned sound cue in a different context. Data are expressed as mean ± SD of 7–8 mice for each treated group and analyzed with one-way ANOVA test followed by Bonferroni multiple comparison test. ^∗^*P* < 0.05 and ^∗∗^*P* < 0.01 vs. Con group; ^&^*P* < 0.05 vs. Anes group; ^#^*P* < 0.05 vs. DM group.

In our previous studies, we found that long-term (6 h) isoflurane inhalation induced hippocampal IR and exacerbated pre-existing hippocampal IR (Fang et al., 2020). Furthermore, both clinical and preclinical studies have found that hippocampal IR is associated with cognitive decline (Biessels and Reagan, 2015; Grillo et al., 2015). We investigated whether long-term isoflurane inhalation also induces cognitive deficits in adult mice and aggravates cognitive dysfunction in T2DM mice. In adult wild type (WT) mice, 6 h of isoflurane anesthesia (Anes group) significantly reduced contextual fear memory as measured by freezing (%) compared to control mice (Con group) from Days 1 to 7 post-treatment (Day 1: 68.96 ± 7.32% vs. 84.70 ± 10.95%, *P* = 0.034; Day 3: 62.14 ± 7.04% vs. 87.97 ± 7.52%, *P* < 0.001; Day 7: 57.88 ± 7.04% vs. 87.97 ± 7.52%, *P* = 0.004), indicating disruption of hippocampal function (Figure 2A). In T2DM model mice as well, isoflurane exposure (DM + Anes group) significantly reduced freezing to context compared to controls (DM group) from Days 1 to 7 (Day 1: 52.73 ± 12.27% vs. 69.66 ± 7.27%, *P* = 0.02; Day 3: 50.75 ± 9.83% vs. 64.33 ± 10.44%, *P* = 0.028; Day 7: 52.77 ± 9.70 vs. 69.43 ± 7.54%, *P* = 0.046). Compared to the Anes group, DM + Anes mice demonstrated even lower freezing on Day 1 (68.96 ± 7.32% vs. 52.73 ± 12.27%, *P* = 0.027) and Day 3 (64.17 ± 7.04% vs. 50.75 ± 9.83%, although the difference did not reach significance, *P* = 0.092), suggesting that pre-existing IR and isoflurane synergistically impair hippocampal function (Figure 2A). By Day 14, no significant differences in contextual fear conditioning were observed in any group. In contrast, there were no significant effects on cued fear conditioning, suggesting that these effects are selective for hippocampal function (Figure 2B).

### Metformin Alleviates Hippocampal IR and Cognitive Dysfunction Caused by Long-Term Isoflurane Inhalation

**FIGURE 3:**
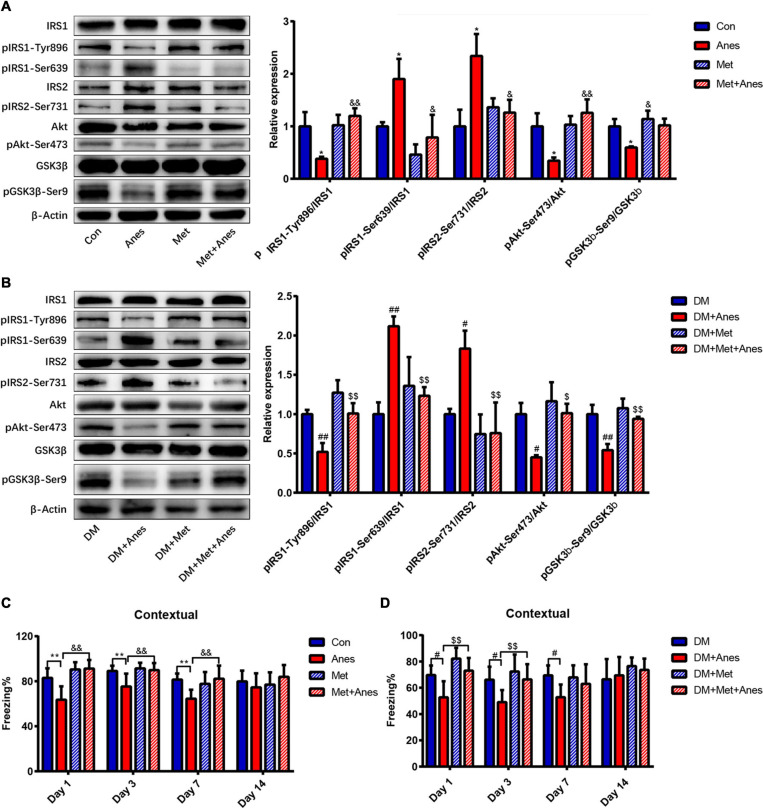
Metformin alleviates hippocampal insulin resistance and impairs contextual fear memory induced by long-term isoflurane anesthesia, but not diabetic pathology. **(A,B)** Representative western blots (left panels) and densitometric analysis (right panels) showing the effects of isoflurane anesthesia, metformin, and anesthesia plus metformin pretreatment on the protein expression levels of hippocampal insulin-signaling pathway components in adult WT mice **(B)** and T2DM mice **(C)** (*n* = 3 mice per group). **(C)** Freezing time to context by adult WT mice receiving vehicle (Con), anesthesia (Anes), metformin (Met, 50 mg/kg), or metformin before anesthesia (Met + Anes) (*n* = 7–8). **(D)** Freezing time to context by T2DM mice receiving vehicle (DM), anesthesia (DM + Anes), metformin (DM + Met, 50 mg/kg), or metformin before anesthesia (DM + Met + Anes) (*n* = 7–8 mice per group). Data are expressed as mean ± SD and analyzed with one-way ANOVA test followed by Bonferroni multiple comparison test. ^∗^*P* < 0.05 and ^∗∗^*P* < 0.01 vs. Con group; ^&^*P* < 0.05 and ^&⁣&^*P* < 0.01 vs. Anes group; ^#^*P* < 0.05 and ^##^*P* < 0.01 vs. DM group; ^$^*P* < 0.05 and ^$$^*P* < 0.01 vs. DM + Anes group.

Decrease insulin resistance and increased insulin sensitivity are the main mechanisms underlying the therapeutic effects of metformin (Giannarelli et al., 2003), so we examined changes in the insulin signaling pathway components insulin receptor substrates 1 and 2 (IRS1 and IRS2), Akt, and GSK-3β among adult WT mice and T2DM mice intraperitoneally pretreated with metformin 50 mg/kg or vehicle 1 h before long-term isoflurane or control inhalation. In adult WT mice, metformin alone had no effect on the mRNA and protein expression levels of IRS1, IRS2, Akt, and GSK-3b (Supplementary Figure 1, all the *P* < 0.05). The phosphorylation levels of IRS1 at Ser639 (pIRS1-Ser639) and IRS2 at Ser731 (pIRS2-Ser731) were upregulated significantly after long-term isoflurane inhalation (Anes group) compared to controls (Con group) (pIRS1-Ser639 1.90 ± 0.38% vs. 1.00 ± 0.08%, *P* = 0.043; pIRS2-Ser731: 2.34 ± 0.42% vs. 1.00 ± 0.32% *P* = 0.033), and these effects were significantly suppressed by intraperitoneal metformin (pIRS1-Ser639 down to 0.79 ± 0.43%, *P* = 0.013; pIRS2-Ser731 down to 1.26 ± 0.24%, *P* = 0.035) (Figure 3A). In contrast, long-term isoflurane inhalation reduced phosphorylation of IRS1 at Tyr896 (pIRS1-Tyr896 0.38 ± 0.04% vs. 1.00 ± 0.27%, *P* = 0.020), phosphorylation of Akt at Ser473 (pAkt-Ser473, 0.34 ± 0.06% vs. 1.00 ± 0.25, *P* = 0.023), and phosphorylation of GSK-3β at Ser9 (pGSK-3β-Ser9, 0.60 ± 0.02% vs. 1.00 ± 0.14, *P* = 0.023), while pretreatment with metformin significantly reversed the downregulation of pIRS1-Tyr896 (to 1.20 ± 0.15%, *P* = 0.004), pAkt-Ser473 (to 1.26 ± 0.26%, *P* = 0.003), and pGSK-3β-Ser9 (to 1.02 ± 0.12%, *P* = 0.018) (Figure 3A). Compared with HFD/STZ induced T2DM mice (DM group), isoflurane also induced the upregulation of pIRS1-Ser639 (1.00 ± 0.15% vs. 2.12 ± 0.13%, *P* = 0.001) and pIRS2-Ser731 (1.00 ± 0.07% vs. 1.83 ± 0.23%, *P* = 0.027), as well as the downregulation of pIRS1-Tyr896 (1.00 ± 0.05% vs. 0.52 ± 0.11%, *P* = 0.007), pAkt-Ser473 (1.00 ± 0.14% vs. 0.45 ± 0.03%, *P* = 0.013), and pGSK-3β-Ser9 (1.00 ± 0.12% vs. 0.54 ± 0.08%, *P* = 0.002) in T2DM mice (Figure 3B). Compared to vehicle pretreatment before isoflurane (DM + Anes group), metformin pretreatment (DM + Met + Anes group) reversed the upregulation of pIRS1-Ser639 (to 1.23 ± 0.11%, *P* = 0.006) and pIRS2-Ser731 (to 0.76 ± 0.39%, *P* = 0.006), induced by isoflurane, as well as the downregulation of pIRS1-Tyr896 (to 1.01 ± 0.13%, *P* = 0.007), pAkt-Ser473 (to 1.01 ± 0.12%, *P* = 0.012), and pGSK-3β-Ser9 (to 0.94 ± 0.03%, *P* = 0.005) in T2DM mice (Figure 3B). Although we previously demonstrated that induction of diabetic pathology by HFD/STZ treatment also markedly induced hippocampal IR (Fang et al., 2020), metformin treatment (DM + Met) failed to activate hippocampal insulin signaling pathways in T2DM mice (all *P* > 0.05).

In addition to normalizing hippocampal insulin signaling following isoflurane anesthesia among adult WT mice, intraperitoneal metformin treatment 1 h before anesthesia (Met + Anes group) also alleviated the impairment in contextual fear memory observed in the Anes group on Day 1 (91.20 ± 7.74% vs. 63.58 ± 11.91%, *P* < 0.001), Day 3 (89.93 ± 6.19% vs. 75.35 ± 11.40%, *P* = 0.004) and Day 7 (82.24 ± 11.64% vs. 64.46 ± 8.04%, *P* = 0.004) (Figure 3C). Similarly, in T2DM mice, isoflurane inhalation (DM + Anes group) decreased freezing time to context compared to untreated controls (DM group) on Day 1 (52.73 ± 12.27% vs. 69.66 ± 7.27%, *P* = 0.017), Day 3 (49.03 ± 9.23% vs. 66.12 ± 9.87%, *P* = 0.047), and Day 7(52.77 ± 9.70% vs. 69.43 ± 7.54%, *P* = 0.033), while metformin pretreatment (DM + Met + Anes group) increased freezing time compared to DM + Anes group mice on Day 1 (73.05 ± 0.61% vs. 52.73 ± 12.27%, *P* = 0.003) and Day 3 (66.34 ± 11.60% vs. 49.03 ± 9.23%, *P* = 0.034), but had no effect on Day 7 (Figure 3D). Further, there were no significant differences in cued freezing among T2DM mice (Supplementary Figure 2B, all the *P* > 0.05).

### Metformin Inhibits Hippocampal Tau Hyperphosphorylation and Increases NR2B Tyr1472 Phosphorylation in Isoflurane-Anesthetized Mice

**FIGURE 4:**
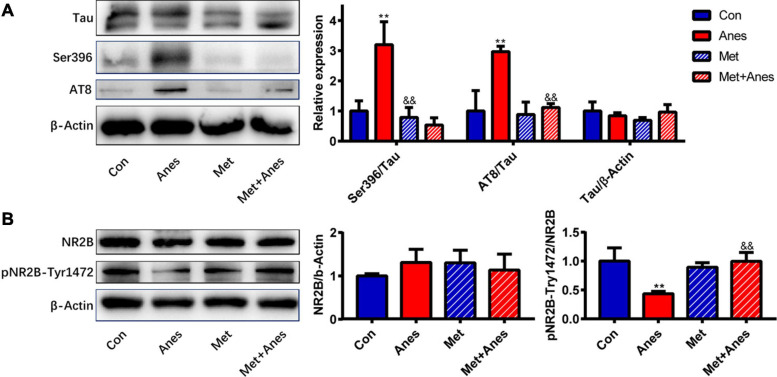
Metformin alleviates tau hyperphosphorylation and pNR2B-Tyr1472 downregulation caused by long-term isoflurane anesthesia. Representative western blots (left panels) and densitometric analysis (right panels, *n* = 3 mice per group) showing the effects of isoflurane anesthesia (Anes group), metformin (Met group), and anesthesia plus metformin pretreatment (Met + Anes group) on expression levels of total tau (Tau) and phosphorylated tau (Ser396 and AT8) **(A)**, as well as total NR2B (NR2B) and phosphorylated NR2B at Tyr1472 (pNR2B-Tyr1472) **(B)** in the hippocampus. β-Actin was used as a gel loading control. Data are expressed as mean ± SD and compared by one-way ANOVA followed by Bonferroni multiple comparisons tests. ^∗∗^*P* < 0.01 compared to the Con group; ^&⁣&^*P* < 0.01 compared to the Anes group.

Clinical and preclinical studies have found that impaired insulin signaling induces tau hyperphosphorylation and that insulin-sensitizing strategies improve cerebrospinal fluid biomarkers of tauopathy in patients with MCI/AD (Hölscher, 2014; Yarchoan et al., 2014; Kim et al., 2015). As a cytoskeleton-associated protein, tau plays an important role in postsynaptic targeting of the Src family tyrosine kinase Fyn to NMDA receptors and in phosphorylating the NR2B subunit at Tyr1472 (pNR2B-Tyr1472), which is associated with NMDA receptor-dependent plasticity (Ittner et al., 2010). Conversely, hyperphosphorylated tau was shown to reduce Fyn binding (Reynolds et al., 2008), suggesting that tau hyperphosphorylation may decrease the expression of hippocampal pNR2B-Tyr1472 after long-term isoflurane anesthesia. Our previous studies indeed found that isoflurane inhalation inhibited hippocampal pNR2B-Tyr1472 expression and that this response was associated with cognitive decline3 (Xia et al., 2016). We further examined whether metformin can alleviate hippocampal IR and improve both tau hyperphosphorylation and pNR2B-Tyr1472 downregulation caused by long-term isoflurane anesthesia. Isoflurane (Anes group) increased tau phosphorylation at Ser396 (pTau-Ser396) and AT8 compared to the Con group (pTau-Ser396: 3.20 ± 0.76% vs. 1.00 ± 0.34, *P* = 0.002; AT8: 2.970 ± 0.18% vs. 1.00 ± 0.68, *P* = 0.002) (Figure 4A). Conversely, isoflurane reduced hippocampal pNR2B-Tyr1472/NR2B compared to the Con group (0.44 ± 0.04% vs. 1.00 ± 0.23%, *P* = 0.008) (Figure 4B). Pretreatment with metformin (Met + Anes group) reversed the increase in hippocampal pTau-Ser396/Tau and AT8/Tau compared to isoflurane alone (pTau-Ser396: 0.53 ± 0.24% vs. 3.20 ± 0.76%, *P* = 0.001; AT8: 1.12 ± 0.13% vs. 2.970 ± 0.18%, *P* = 0.003), as well as the isoflurane-induced reduction in hippocampal pNR2B-Tyr1472/NR2B (1.00 ± 0.15% vs. 0.44 ± 0.04%, *P* = 0.009), while metformin alone (Met group) had no effect on the expression levels of hippocampal pTau-Ser396/Tau, AT8/Tau and pNR2B-Tyr1472 compared to the Con group (Figure 4).

### Glycemic Control Did Not Contribute to the Effects of Metformin on Contextual Fear Memory

**FIGURE 5:**
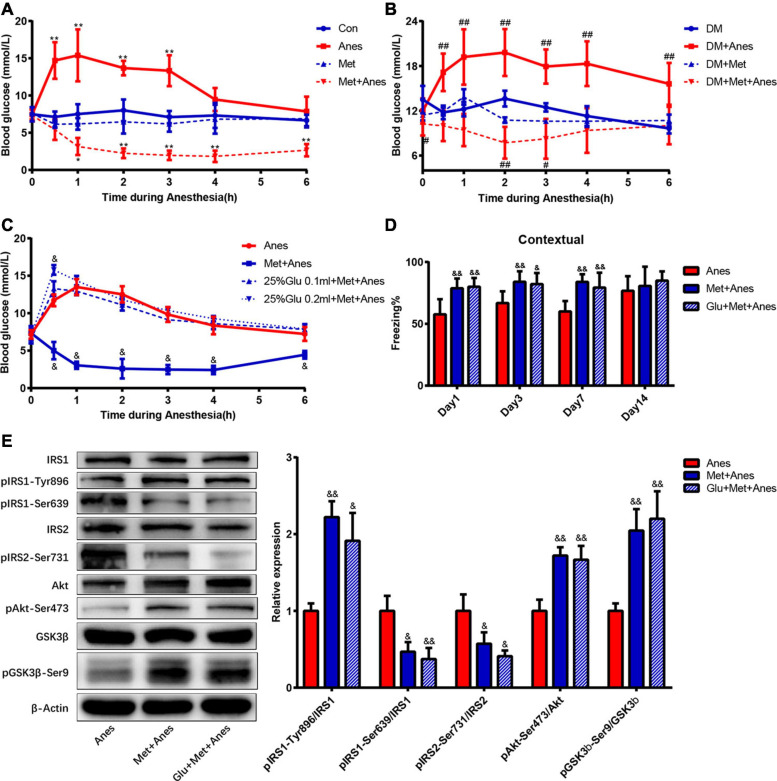
Metformin improves hippocampal IR and contextual fear memory, independent of antiglycemic effects. **(A,B)** Changes in blood glucose levels during anesthesia among adult WT mice **(A)** and T2DM mice **(B)** with or without metformin pretreatment (*n* = 4–6). **(C)** Changes in blood glucose levels during isoflurane anesthesia among WT mice receiving pretreatment with vehicle (Anes), metformin (Met + Anes), 25% glucose 0.1 ml plus metformin (25% Glu 0.1 ml + Met + Anes), or 25% glucose 0.2 ml plus metformin (25% Glu 0.2 ml + Met + Anes) (*n* = 4 for each group). Blood glucose levels are expressed as mean ± SD and compared by repeated measures ANOVA followed by Bonferroni multiple comparisons tests. Repeated measures ANOVA revealed a significant effect of treatment in adult WT mice [*F*_(__3_,_18__)_ = 84.091, *P* < 0.001] and T2DM mice [*F*_(__3_,_13__)_ = 30.604, *P* < 0.001], as well as a treatment × time interaction [WT mice: *F*_(__18_,_45__)_ = 3.281, *P* = 0.001; T2DM mice: *F*_(__18_,_30__)_ = 2.149, *P* < 0.001]. There was also a marked effect of time in WT mice [*F*_(__6_,_13__)_ = 8.552, *P* = 0.001], but not T2DM mice [*F*_(__6_,_8__)_ = 2.534, *P* = 0.112]. Simple contrasts indicated administration of 25% glucose 0.1 ml or 0.2 ml to metformin-pretreated mice induced obvious hyperglycemia (both *P* < 0.001) and no substantial differences in blood glucose levels among Anes, 25%Glu 0.1 ml + Met + Anes, and 25%Glu 0.2 ml + Met + Anes group mice (all *P* > 0.05). **(D)** Freezing times to context by adult WT mice receiving vehicle, metformin, or 25% glucose 0.1 ml plus metformin (Glu + Met + Anes) prior to isoflurane anesthesia (*n* = 7–8). **(E)** Representative western blots (left panels) and densitometric analysis (right panels, *n* = 3 for each group) showing the effects of vehicle, metformin, and 25% glucose 0.1 ml plus metformin on the protein expression levels of hippocampal insulin signaling components in isoflurane anesthetized adult WT mice. Data of freezing conditional test and Western blots are expressed as mean ± SD and analyzed with one-way ANOVA test followed by Bonferroni multiple comparison test. ^∗^*P* < 0.05 and ^∗∗^*P* < 0.01 vs. Con group, ^#^*P* < 0.05 and ^##^*P* < 0.01 vs. DM group. ^&^*P* < 0.05 and ^&⁣&^*P* < 0.01 vs. Anes group.

Previous studies have shown that intraoperative hyperglycemia is associated with increased risk of postoperative cognitive dysfunction, and our previous studies found that long-term isoflurane increased blood glucose in adult WT and T2DM mice (Puskas et al., 2007; Fang et al., 2020). Thus, metformin may alleviate cognitive decline by reducing anesthesia-induced hyperglycemia. To examine this possibility, we compared blood glucose levels among adult WT and T2DM mice receiving anesthesia alone, metformin alone, and anesthesia plus metformin pretreatment with or without glucose supplementation. Repeated measures ANOVA with treatments (Con, Anes, Met and Met + Anes) and time as within-subject factors revealed a significant effect of treatment in adult WT mice [*F*_(__3_,_18__)_ = 84.091, *P* < 0.001] and T2DM mice [*F*_(__3_,_13__)_ = 30.604, *P* < 0.001], as well as a treatment × time interaction (Adult mice: [*F*_(__18_,_45__)_ = 3.281, *P* = 0.001]; T2DM mice: [*F*_(__18_,_30__)_ = 2.149, *P* < 0.001] (Figures 5A,B). There was also a marked effect of time in adult WT mice [*F*_(__6_,_13__)_ = 8.552, *P* = 0.001], but not T2DM mice [*F*_(__6_,_8__)_ = 2.534, *P* = 0.112] (Figures 5A,B). Simple contrasts indicated that blood glucose levels were significantly higher in Anes group mice compared to Con mice (*P* < 0.001), whereas Met + Anes mice had lower blood glucose levels than Con, Anes, and Met group mice (all the *P* < 0.001). Isoflurane anesthesia upregulated blood glucose levels in adult WT mice at 0.5 h (14.70 ± 2.46 mmol/L vs. 7.12 ± 0.72 mmol/L, *P* < 0.001), 1 h (15.38 ± 3.50 mmol/L vs. 7.50 ± 1.34 mmol/L, *P* < 0.001), 2 h (13.68 ± 0.97 mmol/L vs. 8.00 ± 1.46 mmol/L, *P* < 0.001), and 3 h (13.33 ± 2.08 mmol/L vs. 7.10 ± 0,83 mmol/L, *P* < 0.001), while intraperitoneal pretreatment with metformin 50 mg/kg 1 h before anesthesia not only completely reversed the hyperglycemic impact of isoflurane but also further decreased blood glucose levels compared to the Con group at 1 h (3.15 ± 1.15 mmol/L vs. 7.50 ± 1.34 mmol/L, *P* = 0.017)–6 h (2.65 ± 0.82 mmol/L vs. 6.67 ± 0.77 mmol/L, *P* < 0.001) (Figure 5A). A similar glycemic effect of isoflurane was also found in T2DM mice, but was much longer lasting [from 0.5 h (17.15 ± 2.51 mmol/L vs. 11.78 ± 0.92 mmol/L, *P* < 0.001) to 6 h (15.60 ± 2.80 mmol/L vs. 9.64 ± 0.67 mmol/L, *P* < 0.001)]. Although metformin still reversed the glycemic impact of isoflurane (*P* < 0.001) in T2DM model mice and decreased blood glucose levels compared to control T2DM mice (*P* = 0.019), the duration was much shorter than in adult WT mice (0 h: 10.24 ± 1.55 mmol/L vs. 13.50 ± 1.83 mmol/L, *P* = 0.018; 2 h: 7.72 ± 2.13 mmol/L vs. 13.62 ± 1.06 mmol/L, *P* = 0.003; 3 h: 8.24 ± 2.67 mmol/L vs. 12.45 ± 0.56 mmol/L, *P* = 0.031). The blood glucose levels of Met and DM + Met groups were generally lower than in Con and DM groups, respectively, but the differences did not reach statistical significance (Figure 5B).

To eliminate the contribution of glycemic control to metformin effects on contextual fear memory, 0.1 or 0.2 ml 25% glucose solution was given by gavage before anesthesia. Simple contrasts indicated administration of 25% glucose 0.1 or 0.2 ml to metformin-pretreated mice induced obvious hyperglycemia (both *P* < 0.001) and no substantial differences in blood glucose levels among Anes, 25%Glu 0.1 ml + Met + Anes, and 25%Glu 0.2 ml + Met + Anes group mice (all *P* > 0.05). Moreover, blood glucose levels did not differ between Anes and 25%Glu 0.1 ml + Met + Anes groups at any time point during anesthesia (Figure 5C). Thus, administration of 25% glucose solution essentially eliminated the antiglycemic effect of metformin. However, metformin pretreatment still markedly improved hippocampal insulin resistance in WT mice also receiving glucose prior to anesthesia (25%Glu 0.1 ml + Met + Anes group) compared to anesthesia alone (Anes group) (pIRS1-Tyr896: 1.91 ± 0.36% vs. 1.00 ± 0.10%, *P* = 0.012; pIRS1-Ser639: 0.37 ± 0.14% vs. 1.00 ± 0.20%, *P* = 0.009; pIRS2-Ser731: 0.41 ± 0.07% vs. 1.00 ± 0.21%, *P* = 0.011, pAkt-Ser47: 1.67 ± 0.18% vs. 1.00 ± 0.15%, *P* = 0.004; pGSK-3β-Ser9: 2.20 ± 0.36% vs. 1.00 ± 0.10%, *P* = 0.005). Moreover, metformin still enhanced contextual fear memory compared to the Anes group on Day 1 (57.67 ± 12.26% vs. 79.99 ± 7.14%, *P* = 0.001), Day 3 (66.79 ± 9.51% vs. 82.17 ± 8.94%, *P* = 0.015), and Day 7 (60.00 ± 8.44% vs. 79.31 ± 12.04%, *P* = 0.003) (Figure 5E). In addition, neither hippocampal insulin signing nor contextual fear memory differed between 25%Glu 0.1 ml + Met + Anes and control mice (Figure 5D), indicating complete reversal of anesthesia-induced IR and memory impairment even when the antiglycemic effect was blocked.

## Discussion

Our present study demonstrated that prolonged (6 h) isoflurane anesthesia can induce hippocampal insulin resistance and impair hippocampus-dependent contextual fear memory in adult WT mice. Further, these deleterious effects were exacerbated in HDF/STZ-induced T2DM model mice. Intraperitoneal pretreatment with metformin (50 mg/kg body weight) attenuated isoflurane-induced hippocampal IR and contextual fear memory impairment in WT mice, but had a more blunted effect in T2DM model mice. Metformin also reduced hippocampal tau hyperphosphorylation and increased expression of hippocampal pNR2B-Tyr1472, which may have contributed to the improvement in hippocampus-dependent memory. Metformin also successfully reversed isoflurane-induced hyperglycemia, but elimination of glycemic control by high oral glucose did not influence the effects on IR and memory impairment, suggesting a novel independent mechanism for the therapeutic benefits of this antidiabetic drug.

### Isoflurane and HFD/STZ Synergistically Induce Hippocampus-Dependent Cognitive Decline

As T2DM is one of the most common, costly, and disabling conditions in the industrialized world (Cho et al., 2018), we focused on the role of preoperative IR created by HFD/STZ treatment and postoperative IR caused by long-term isoflurane inhalation on POCD. The combination of HFD feeding and low-dose STZ treatment is a classic method for inducing a T2DM-like phenotype in mice (Srinivasan et al., 2005). Indeed, our previous study demonstrated that HFD/STZ could induce marked peripheral and brain IR in mice (Fang et al., 2020). Brain insulin action is required for neuronal survival, synaptic plasticity, and cognitive function (van der Heide et al., 2006; Biessels and Reagan, 2015; Grillo et al., 2015). Both adult WT mice and T2DM mice demonstrated impairments in contextual fear conditioning after long-term isoflurane inhalation. However, isoflurane had no effects on cued fear conditioning. While the amygdala is critical for the expression of both cued and contextual conditioned fear responses, contextual fear memories are consolidated and maintained by the hippocampus (Chaaya et al., 2018). Thus, isoflurane and HFD/STZ treatment appear to selectively impair hippocampus-dependent fear learning and memory. Compared to adult WT mice receiving anesthesia (Anes group), T2DM model mice (DM + Anes group) displayed significantly weaker context-dependent freezing responses on Day 1 and a very weak freezing response on Day 3 that did not differ after long-term isoflurane inhalation, suggesting that contextual fear learning and memory were more severely impaired in T2DM mice. This is consistent with clinical studies reporting that diabetes patients are more susceptible to POCD (Feinkohl et al., 2017). Thus, preoperative IR and postoperative IR have synergistic effects on POCD.

### Metformin Attenuated Hippocampal IR and Tau Hyperphosphorylation and Increase Hippocampal pNR2B-Tyr1472 Expression

To examine potential mechanisms underlying the memory impairments induced by isoflurane and HFD/STZ treatment, we measured the effects of the first-line antidiabetic drug metformin on cognitive performance, IR, and phosphorylation of the NMDA receptor, which is essential for many forms of synaptic plasticity underlying hippocampus-dependent learning and memory (Rostas et al., 1996; Petrone et al., 2003). Metformin has been shown to cross the blood-brain barrier rapidly and effectively ameliorate IR (Giannarelli et al., 2003; Łabuzek et al., 2010). Increases in IRS1 and IRS2 serine phosphorylation, as well as decreases in downstream Akt and GSK3β phosphorylation are hallmark features of brain IR (Fang et al., 2020). Consistent with hippocampal IR, long-term isoflurane inhalation increased pIRS1-Ser639 and pIRS2-Ser731 expression levels and downregulated pIRS1-Tyr896, pAkt-Ser473, and pGSK3β-Ser9 expression levels in the hippocampus, while metformin reversed these responses. A similar pattern of changes was also observed in long-term isoflurane anesthetized T2DM mice. Concomitant with improved hippocampal IR, metformin pretreatment ameliorated the impairment in fear learning induced by long-term isoflurane inhalation in adult WT mice, but was less effective in T2DM mice. These results are consistent with numerous studies implicating hippocampal IR as a key mediator of cognitive dysfunction (Reviewed in Biessels and Reagan, 2015). Clinical and preclinical studies have also revealed that impaired insulin signaling induces tau hyperphosphorylation and that insulin-sensitizing strategies improve cerebrospinal fluid biomarkers of disease in patients with mild cognitive impairment or AD (Hölscher, 2014; Yarchoan et al., 2014; Kim et al., 2015). Consistent with these clinical findings, isoflurane upregulated the expression of p-tau in hippocampus, while metformin decreased the expression of hippocampal p-tau. As a cytoskeleton-associated protein, tau plays an important role in the postsynaptic targeting of the Scr kinase Fyn to NMDA receptors, where it phosphorylates the NR2B subunit at Tyr-1472 and promotes NMDAR-dependent synaptic plasticity (Rostas et al., 1996; Petrone et al., 2003; Ittner et al., 2010). Moreover, hyperphosphorylated tau has been shown to reduce Fyn binding (Reynolds et al., 2008). Metformin not only attenuated IR, but also inhibited the phosphorylation of tau protein and prevented isoflurane-induced downregulation of pNR2B-Tyr-1472, providing a feasible mechanism for the improvement in contextual fear memory following metformin treatment. Therefore, metformin pretreatment may effectively alleviate hippocampal IR, prevent tau hyperphosphorylation, enhance the phosphorylation of Tyr-1472 in the NR2B, and reverse cognitive impairment caused by long-term isoflurane inhalation.

### Metformin Mitigated Cognitive Decline Through a Mechanism Independent of Glycemic Control

Previous studies have shown that hyperglycemia contributes to cognitive decline in diabetes patients (Cox et al., 2005). Higher glycated hemoglobin, an index of more severe and prolonged hyperglycemia, is associated with a greater magnitude of cognitive impairment, and even acute hyperglycemia can impair cognitive performance in persons with type 1 or type 2 diabetes mellitus (Sommerfield et al., 2004; Gao et al., 2015; Šuput Omladič et al., 2020). Intraoperative hyperglycemia also increases the risk of POCD (Puskas et al., 2007; Schricker et al., 2014). Our previous animal studies and related clinical studies have shown that isoflurane inhalation significantly increases blood glucose (Behdad et al., 2014; Fang et al., 2020). In addition to relieving insulin resistance, metformin reduces blood sugar by suppressing hepatic glucose production, increasing glucose uptake into muscle, and promoting the transport of blood glucose into stool (Giannarelli et al., 2003; Morita et al., 2020). Our present studies did find that intraperitoneal injection of metformin (50 mg/kg body weight) not only decreased blood glucose in adult WT and T2DM mice, but also dramatically reversed the hyperglycemic effect of isoflurane during anesthesia. Surprisingly, however, glycemic control did not contribute to the improvement in hippocampus-dependent fear memory under the current experimental conditions, as gavage treatment with 25% glucose solution elevated blood glucose even under metformin treatment, but did not influence metformin-associated improvements in insulin signaling pathways and fear learning following isoflurane anesthesia. The mechanism by which acute hyperglycemia causes cognitive decline remains unclear. Whether hyperglycemia impair cognitive performance through insulin signaling need further study.

### Pretreatment of Metformin Had No Therapeutic Effects on HFD/STZ-Induced T2DM

Our present study found that metformin had no effects on the activation of hippocampal insulin signaling and intraperitoneal injection of metformin also had no effects on cognitive performance of T2DM mice. However, preclinical studies have shown that metformin treatment improved cognitive deficits in db/db mice and chronic HFD-fed mice (Li et al., 2012; Pintana et al., 2012; Chen et al., 2019). Clinical studies also demonstrated that long-term usage can lower the risk of cognitive impairment in older adults with diabetes (Ng et al., 2014). Different research models and drug treatment times may explain this discrepancy. Ramos-Rodriguez and colleagues found that the central pathologies and cognitive impairments differed among diabetes models (Ramos-Rodriguez et al., 2013). Db/db mice may be more amenable to metformin treatment that C57BL/6J mice used in this study and longer-term HFD treatment (12 weeks) was used for T2DM model animals in previous studies (Li et al., 2012; Pintana et al., 2012; Chen et al., 2019). In previous studies, metformin administration time has varied from 21 days or 8 weeks (Li et al., 2012; Pintana et al., 2012; Chen et al., 2019). Clinical studies reported that short-term metformin treatment (36 weeks) had no effect on cognitive performance in diabetes patients, while longer-term treatment (more than 6 years) was beneficial (Abbatecola et al., 2010; Ng et al., 2014). Whether long-term metformin treatment has therapeutic effects on HFD/STZ-induced T2DM mice requires further study.

## Conclusion

Hippocampal IR, tau hyperphosphorylation, and downregulation of pNR2B-Tyr1472 contribute to prolonged isoflurane-induced cognitive dysfunction. Brief metformin treatment can mitigate these effects through a mechanism independent of glycemic control. However, a single intraperitoneal injection of metformin had less therapeutic effects on cognitive decline induced by isoflurane in T2DM mice. A single-dose pretreatment of metformin had no effects on the changes of hippocampal IR and cognitive decline induced by HFD/STZ. Future studies are needed to investigate whether long-term metformin treatment can also prevent HFD/STZ -induced hippocampal IR and cognitive decline.

## Data Availability Statement

The raw data supporting the conclusions of this article will be made available by the authors, without undue reservation.

## Ethics Statement

The studies involving human participants were reviewed and approved by the Medical Ethics Committee of the Affiliated Drum Tower Hospital of Medical Department of Nanjing University. The patients/participants provided their written informed consent to participate in this study. The animal study was reviewed and approved by Laboratory Animal Ethics Committee of Drum Tower Hospital. Written informed consent was obtained from the individual(s) for the publication of any potentially identifiable images or data included in this article.

## Author Contributions

XGu, TX, and ZM conceived the original idea. FX, XF, and SL performed the animal experiments. FX, XGo, and XF carried out the clinical study. LP, YQ, and XZ generated and analyzed data and wrote the manuscript with input from all authors. All authors read and approved the final manuscript.

## Conflict of Interest

 The authors declare that the research was conducted in the absence of any commercial or financial relationships that could be construed as a potential conflict of interest.

## Publisher’s Note

All claims expressed in this article are solely those of the authors and do not necessarily represent those of their affiliated organizations, or those of the publisher, the editors and the reviewers. Any product that may be evaluated in this article, or claim that may be made by its manufacturer, is not guaranteed or endorsed by the publisher.

## Supplementary Material

Supplementary Figure 1 Metformin had no effects on the mRNA expression levels of hippocampal insulin-signaling pathway components Real-time quantitative RT-PCR showing the effects of isoflurane anesthesia, metformin, and anesthesia plus metformin pretreatment on the mRNA expression levels of IRS1, IRS2, Akt, and GSK3β in hippocampus (*n* = 3 mice per group).

Supplementary Figure 2 Metformin and blood glucose changes had no effects on the cued fear memory. **(A)** Freezing time to cue by adult WT mice receiving vehicle (Con), anesthesia (Anes), metformin (Met, 50 mg/kg), or metformin before anesthesia (Met + Anes) (*n* = 7–8). **(B)** Freezing time to cue by T2DM mice receiving vehicle (DM), anesthesia (DM + Anes), metformin (DM + Met, 50 mg/kg), or metformin before anesthesia (DM + Met + Anes) (*n* = 7–8 mice per group). **(C)** Freezing time to cue by adult WT mice receiving vehicle (Anes), metformin (Met + Anes), or 25% glucose 0.1 ml plus metformin (Glu + Met + Anes) prior to isoflurane anesthesia (*n* = 7–8). Data are expressed as mean ± SD and analyzed with one-way ANOVA test followed by Bonferroni multiple comparison test.

## Abbreviations

AD: Alzheimer’s disease
Anes: Isoflurane anesthesia
DM: T2DM model mice
Glu 25%: glucose solution
HFD: High-fat diet
IR: Insulin resistance
IRS1: Insulin receptor substrates 1
IRS2: Insulin receptor substrates 2
MCI: Mild cognitive impairment
Met: Metformin injection
MetS: Metabolic syndrome
POCD: Postoperative cognitive dysfunction
STZ: Streptozotocin
T2DM: Type 2 diabetes mellitus
WT: Wild type.
