# Supplementary figures and images for: Amelioration of Hippocampal Insulin Resistance Reduces Tau Hyperphosphorylation and Cognitive Decline Induced by Isoflurane in Mice
Source: Front Aging Neurosci. 2021 Aug 25;13:686506. doi: 10.3389/fnagi.2021.686506 (PMC8425557; doi:10.3389/fnagi.2021.686506)

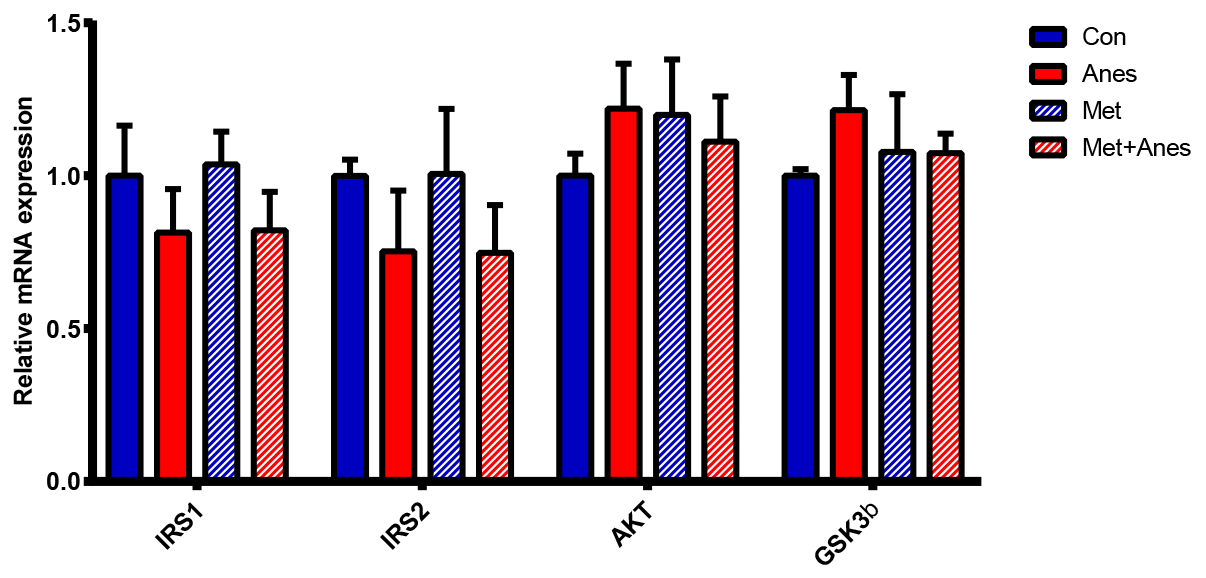

Supplement: Supplementary Figure 1 — Metformin had no effects on the mRNA expression levels of hippocampal insulin-signaling pathway components Real-time quantitative RT-PCR showing the effects of isoflurane anesthesia, metformin, and anesthesia plus metformin pretreatment on the mRNA expression levels of IRS1, IRS2, Akt, and GSK3β in hippocampus (n = 3 mice per group). [file Image_1.TIF]

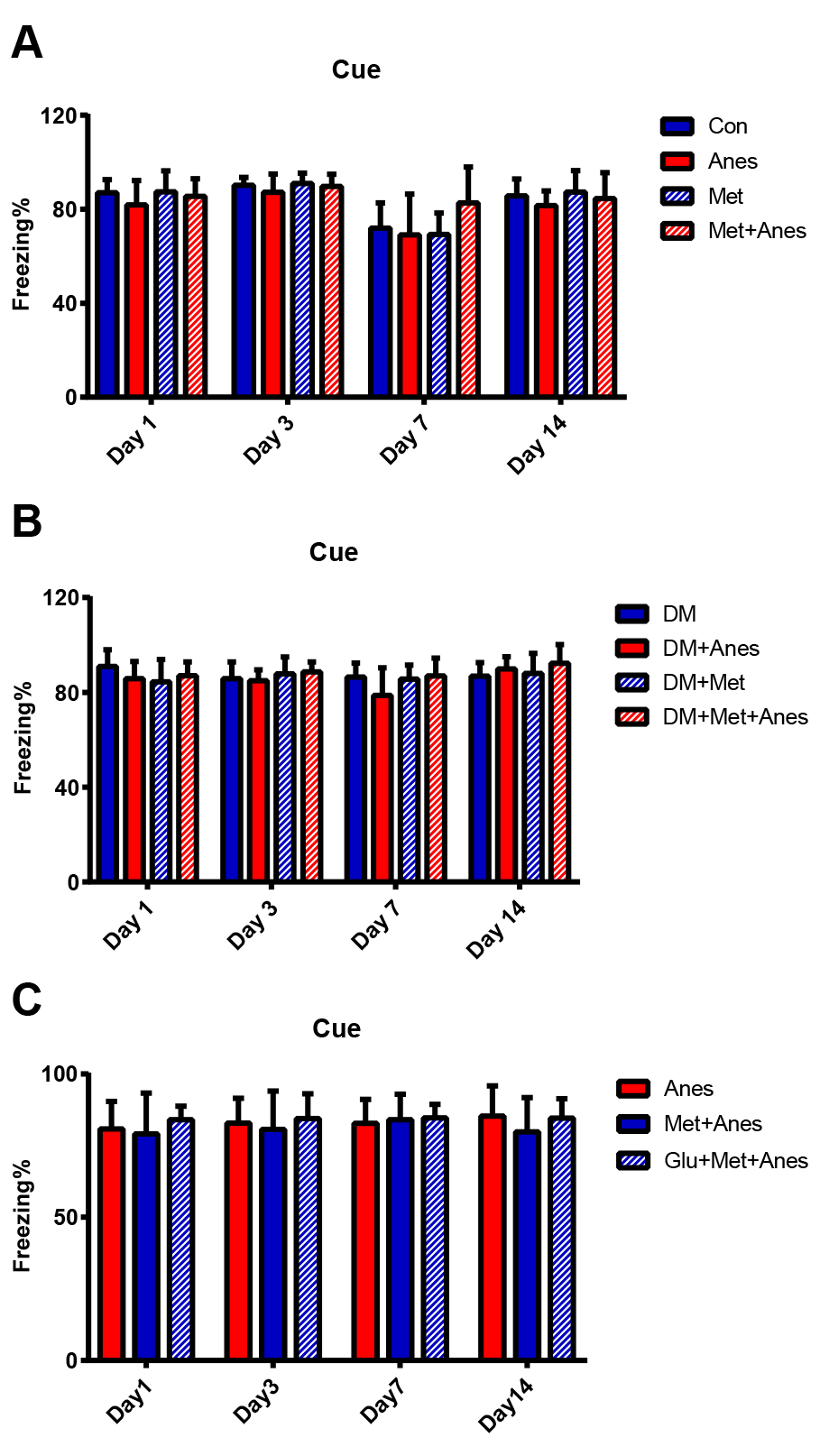

Supplement: Supplementary Figure 2 — Metformin and blood glucose changes had no effects on the cued fear memory. (A) Freezing time to cue by adult WT mice receiving vehicle (Con), anesthesia (Anes), metformin (Met, 50 mg/kg), or metformin before anesthesia (Met + Anes) (n = 7–8). (B) Freezing time to cue by T2DM mice receiving vehicle (DM), anesthesia (DM + Anes), metformin (DM + Met, 50 mg/kg), or metformin before anesthesia (DM + Met + Anes) (n = 7–8 mice per group). (C) Freezing time to cue by adult WT mice receiving vehicle (Anes), metformin (Met + Anes), or 25% glucose 0.1 ml plus metformin (Glu + Met + Anes) prior to isoflurane anesthesia (n = 7–8). Data are expressed as mean ± SD and analyzed with one-way ANOVA test followed by Bonferroni multiple comparison test. [file Image_2.TIF]
