# Supplementary material for: Amelioration of Hippocampal Insulin Resistance Reduces Tau Hyperphosphorylation and Cognitive Decline Induced by Isoflurane in Mice
Source: Front Aging Neurosci. 2021 Aug 25;13:686506. doi: 10.3389/fnagi.2021.686506 (PMC8425557; doi:10.3389/fnagi.2021.686506)
Supplement: Supplementary file 3 [file Table_1.DOCX]

**Table S1. Morbidity of POCD at 1 week after orthopedic surgery**

| **Group** | **POCD** | **Non-POCD** | **Total** | **Morbidity of POCD** | **χ² value** | **P value** |
| --- | --- | --- | --- | --- | --- | --- |
| **Control** | **7** | **49** | **56** | **12.50%** | **3.98** | **0.046** |
| **T2DM** | **14** | **33** | **46** | **28.25%** |  |  |
